# Pulsed resource availability changes dietary niche breadth and partitioning between generalist rodent consumers

**DOI:** 10.1002/ece3.5587

**Published:** 2019-08-20

**Authors:** Ryan B. Stephens, Erik A. Hobbie, Thomas D. Lee, Rebecca J. Rowe

**Affiliations:** ^1^ Natural Resources and the Environment University of New Hampshire Durham NH USA; ^2^ Earth Systems Research Center University of New Hampshire Durham NH USA

**Keywords:** competition, isotopic niche, niche partitioning, optimal foraging, resource pulse

## Abstract

Identifying the mechanisms that structure niche breadth and overlap between species is important for determining how species interact and assessing their functional role in an ecosystem. Without manipulative experiments, assessing the role of foraging ecology and interspecific competition in structuring diet is challenging. Systems with regular pulses of resources act as a natural experiment to investigate the factors that influence the dietary niches of consumers. We used natural pulses of mast‐fruiting of American beech (*Fagus grandifolia*) to test whether optimal foraging or competition structure the dietary niche breadth and overlap between two congener rodent species (*Peromyscus leucopus* and *P. maniculatus*), both of which are generalist consumers. We reconstructed diets seasonally over a 2‐year period using stable isotope analysis (δ^13^C, δ^15^N) of hair and of potential dietary items and measured niche dynamics using standard ellipse area calculated within a Bayesian framework. Changes in niche breadth were generally consistent with predictions of optimal foraging theory, with both species consuming more beechnuts (a high‐quality food resource) and having a narrower niche breadth during masting seasons compared to nonmasting seasons when dietary niches expanded and more fungi (a low‐quality food source) were consumed. In contrast, changes in dietary niche overlap were consistent with competition theory, with higher diet overlap during masting seasons than during nonmasting seasons. Overall, dietary niche dynamics were closely tied to beech masting, underscoring that food availability influences competition. Diet plasticity and niche partitioning between the two *Peromyscus* species may reflect differences in foraging strategies, thereby reducing competition when food availability is low. Such dietary shifts may have important implications for changes in ecosystem function, including the dispersal of fungal spores.

## Graphical abstract

Our study tests the opposing predictions of optimal foraging theory and competition theory for dietary niche breadth and overlap of two closely related and ecologically similar rodent species. We find that the mechanism that structures niche dynamics varies under natural pulses of resource availability with changes in niche breadth consistent with predictions of optimal foraging theory and niche overlap consistent with predictions of competition theory.

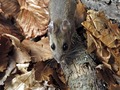

## 1 INTRODUCTION

Systems with pulsed resource availability experience a natural manipulation of high‐quality food resources (Yang, Bastow, Spence, & Wright, 2008) and offer an opportunity to investigate the mechanisms that structure the niches of species (Correa & Winemiller, 2014; Selva, Hobson, Cortés‐Avizanda, Zalewski, & Donázar, 2012; Stapp & Polis, 2003). In terrestrial ecosystems, one of the most common resource pulses is masting (or mast‐fruiting), in which trees of the same species synchronously produce large seed crops in the same season, followed by an extremely low crop the next year (Ostfeld & Keesing, 2000). For consumers, particularly rodents, masting events produce a food source that is not only highly abundant and energy‐rich, but also easily harvested, stored, and defended (Cramer, 2014; Vander Wall, 2010). During nonmasting years, rodents that would otherwise consume seeds must find alternative food sources, such as fungi, which although readily available are relatively low in nutrient content (Cork & Kenagy, 1989; Fletcher et al., 2010).

**Figure 1:**
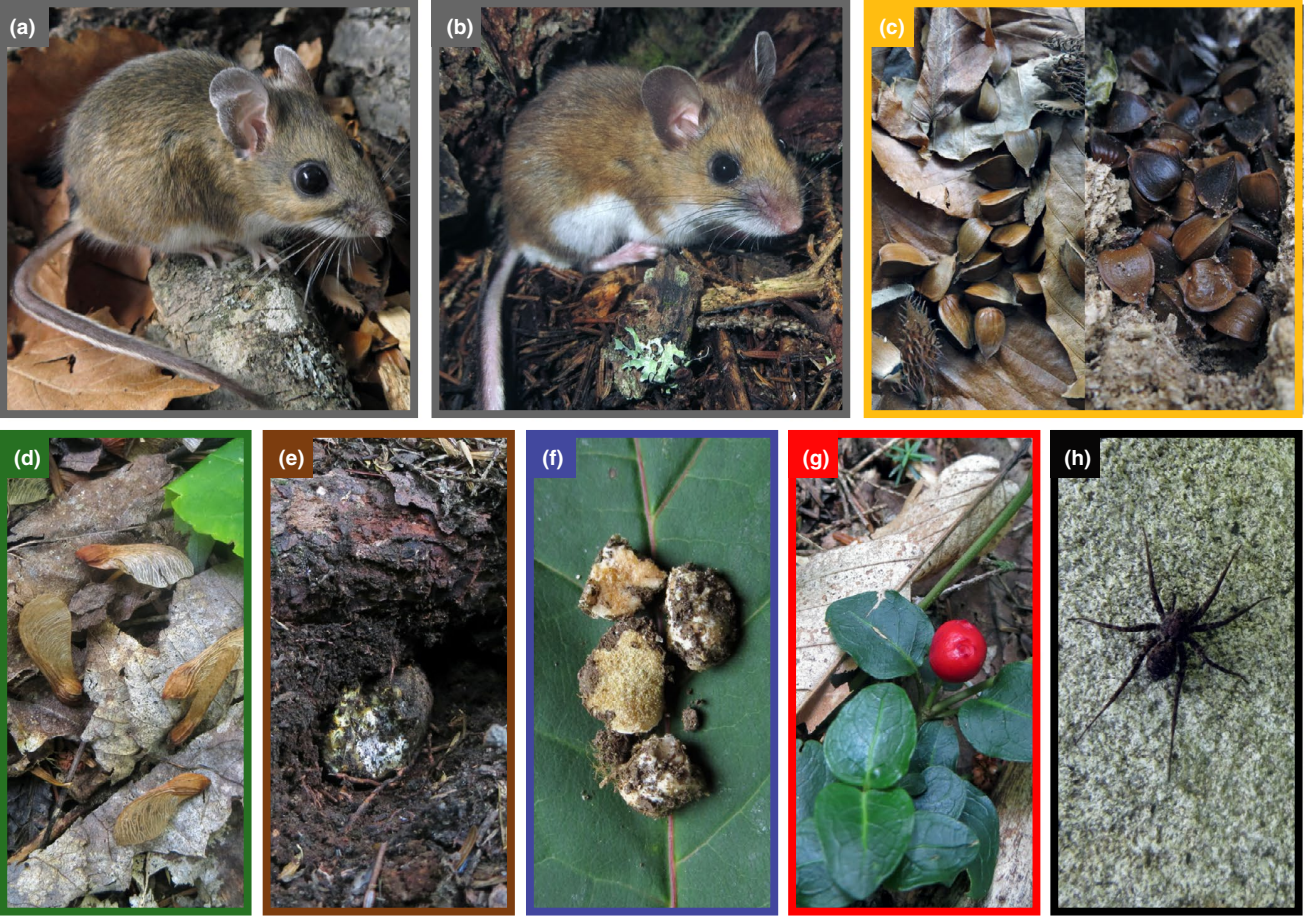
White‐footed mice (a; *Peromyscus leucopus*) and woodland deer mice (b; *P. maniculatus*) are generalist rodents that occur syntopically in forests throughout midwestern and eastern North America. For *Peromyscus*, beechnuts are a high‐quality food source that is abundant and both easy to collect and store during masting periods (c; left—beechnuts dropped during a masting event, right—beechnuts cached in a hollow log). Other available, but ostensibly lower quality, food items for *Peromyscus* include: red maple seeds (d; *Acer rubrum*), ectomycorrhizal truffles (e; excavated and partially consumed sporocarp of *Elaphomyces macrosporus*), arbuscular mycorrhizal truffles (f; sporocarps of *Glomus* spp.), berries (g; partridge berry—*Mitchella repens*), and arthropods (h; spider—order Araneae). Photographs by Ryan Stephens

The white‐footed mouse (*Peromyscus leucopus noveboracensis*) and woodland deer mouse (*P. maniculatus gracilis*) are abundant rodents that are syntopic throughout forests in midwestern and eastern North America (Figure 1a,b; Wolff, Dueser, & Berry, 1985). Both species increase with masting (Elias, Witham, & Hunter, 2004; Falls, Falls, & Fryxell, 2007) and have long been used as models for studying resource use (Davidson & Morris, 2001; Shaner, Bowers, & Macko, 2007) and competition (Dooley & Dueser, 1990) because they have similar morphology and habitat affinities (Stephens, Anderson, Wendt, & Meece, 2014; Wolff, 1996a). Additionally, *P. leucopus* and *P. maniculatus* use similar food resources in syntopy and are thought to be dietary generalists (Hamilton, 1941; Wolff et al., 1985), although Cramer (2014) found that they have different selection preferences for maple seeds (*Acer* spp.) in captivity. To identify the mechanisms that structure dietary niche breadth and overlap among closely related species, we monitored the seasonal diets of syntopically occurring *P. leucopus* and *P. maniculatus* in a temperate forest that had two masting events of American beech (*Fagus grandifolia*). We used stable isotope analysis of hair to measure intraspecific dietary niche breadth and interspecific dietary niche overlap and to test the predictions of optimal foraging theory (MacArthur & Pianka, 1966; Perry & Pianka, 1997) and competition theory (Abrams, 1983; Macarthur & Levins, 1967).

**Figure 2:**
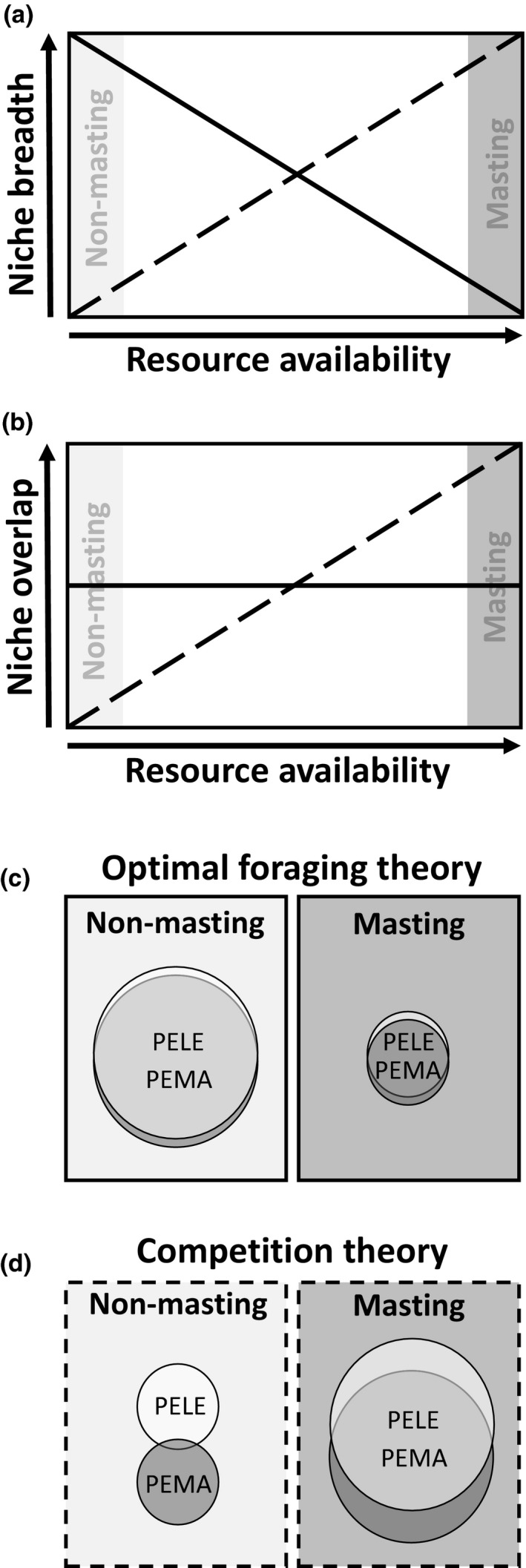
Contrasting predictions for changes in niche breadth (a) and overlap (b) based on optimal foraging theory (solid line; c) and competition theory (dashed line; d) during low resource availability (nonmasting) and high resource availability (masting). Under optimal foraging theory, intraspecific niche breadth is highest during nonmasting (a, c) and interspecific niche overlap for *Peromyscus leucopus* (PELE) and *P. maniculatus* (PEMA) should not differ between nonmasting and masting (b, c). Under competition theory, intraspecific niche breadth and interspecific niche overlap are predicted to be lowest during nonmasting and highest during masting (a, b, d). Note that predictions assume that alternative food sources available during nonmasting times are of low quality and that species do not partition space use

For ecologically similar species that occupy the same space, optimal foraging theory and competition theory generate contrasting predictions of niche dynamics under conditions with pulses of high‐quality food items (Figure 2). Optimal foraging theory states that dietary niche dynamics are driven by the availability of food resources, with individuals choosing food items that maximize their rate of energy intake (MacArthur & Pianka, 1966; Schoener, 1971). When a high‐quality food resource is plentiful, individuals are expected to increase dietary specialization and as availability of this resource declines, individuals become less specialized as they search for alternative food sources (MacArthur & Pianka, 1966). Although optimal foraging theory is generally used to describe the resource use of an individual, these processes also influence niche dynamics of the population. When high‐quality food items are abundant, diets converge and the population niche breadth decreases. When high‐quality food items become scarce, diets become more variable as alternative food sources are consumed, thereby increasing population niche breadth (Schoener, 1971; Stephens & Krebs, 1986; Figure 2a). Optimal foraging theory also predicts that although space use may be influenced by interspecific competition, the food items selected are independent of other species (MacArthur & Pianka, 1966; Figure 2b). In contrast, competition theory predicts that, for ecologically similar species, coexistence is achieved through niche partitioning (Abrams, 1983; Schoener, 1974). Niche partitioning is predicted to be greatest under low resource availability, when species focus on the resource they can best extract, which decreases the diversity of food items in their diets. Niche partitioning subsequently reduces both the dietary niche breadth of the population (Bolnick et al., 2010; Schoener, 1982; Figure 2a) and interspecific dietary similarity or niche overlap (Figure 2b). During times of high resource availability, niche breadths expand and niche overlap can increase because resources no longer limit coexistence (Schoener, 1982; Figure 2a,b).

Based on these assumptions, under optimal foraging theory, we predict that both *Peromyscus* species will show similar patterns of niche overlap, irrespective of masting, and have reduced niche breadths during masting seasons compared to nonmasting seasons (Figure 2c). In contrast, under competition theory, we predict that, relative to nonmasting seasons, both niche overlap and breadth would be high during masting seasons when food resources are not limiting and competition is reduced (Figure 2d).

## 2 MATERIALS AND METHODS

### 2.1 Study system and sample collection

We trapped small mammals and collected food items for isotopic analysis on 12 sampling grids at the Bartlett Experimental Forest, White Mountain National Forest, New Hampshire (44°3′7.2″N, 71°17′25.1″W). Grids were in hardwood (*n* = 4), mixed (*n* = 4), and softwood (*n* = 4) forest stands between 250 and 450 m elevation. Grids consisted of an 8 × 8 station array with 15 m spacing (64 stations; 1.1 ha) and were placed an average of 1.23 km apart (range 0.28–2.61). Hardwood grids were dominated by red maple (*Acer rubrum*) and American beech (*Fagus grandifolia*) with a lesser component of sugar maple (*A. saccharum*), yellow birch (*Betula alleghaniensis*), and white ash (*Fraxinus americana*), whereas softwood grids were dominated by eastern hemlock (*Tsuga canadensis*) and red spruce (*Picea rubens*). Mixed grids were codominated by both hardwood and softwood species. Shrub cover ranged from depauperate to abundant and was primarily composed of hobblebush (*Viburnum lantanoides*); ground cover was lacking except in wet areas where sedges and ferns were common.

Small mammals were captured on each trapping grid using Sherman live traps baited with a bird seed mix and insulated with polyester batting. Traps were set within 1.5 m of each station and checked twice daily (morning and afternoon) for four consecutive days in June, July, and August of 2014 and 2015. This summer trapping was part of a broader study on small mammal ecology. Supplementary trapping was carried out in September or October of both years to collect fall hair samples for isotopic analysis. Captured *Peromyscus* were measured, weighed, sexed, aged (based on pelage color and reproductive status: juvenile, subadult, or adult), and assigned a uniquely numbered ear tag (model 1005‐1; National Band and Tag Company). *Peromyscus leucopus* and *P. maniculatus* were differentiated based on measurements, particularly ear length (Stephens et al., 2014), and questionable individuals were confirmed using genetic analyses. For isotopic analysis, we collected approximately 1–4 mg of hair from the dorsal posterior of an individual upon first capture and only took additional hair samples if molting occurred between trapping periods. We used the number of *Peromyscus* captured on a grid from June to August as a general index of abundance within years. The trapping protocol was approved by the University of New Hampshire Animal Care and Use Committee (protocol 140304) and followed guidelines outlined by the American Society of Mammalogists (Sikes & Animal Care and Use Committee of the American Society of Mammalogists, 1973).

For isotopic analysis of the resource base, we collected six potential food sources known to comprise the majority of dietary items of *P. leucopus* and *P. maniculatus*: beechnuts, red maple seeds, ectomycorrhizal (EM) fungal sporocarps, arbuscular mycorrhizal (AM) fungal sporocarps, berries, and arthropods (Figure 1c–g; Linzey & Linzey, 1973; Wolff et al., 1985). Beechnuts, red maple seeds, and berries (only abundant berry‐producing species on sampling grids: hobblebush and partridge berries [*Mitchella repens*]) were collected opportunistically while trapping. Arthropods were collected using small pitfall traps and were analyzed at the taxonomic rank of order: beetles (Coleoptera), grasshoppers (Orthoptera) and spiders (Araneae). EM sporocarps (genus *Elaphomyces*) were collected as part of a companion study (Stephens, Remick, Ducey, & Rowe, 2017). AM sporocarps are extremely small and were not detected during truffle field surveys (Stephens et al., 2017). Instead, we used *Glomus*, a common sporocarp‐producing AM fungus, which was collected in *Acer*‐dominated forest in Durham, New Hampshire. Individual samples within a food source were aggregated at the grid level to form a composite sample, with the exception of EM and AM fungi for which samples were analyzed individually.

### 2.2 Beech masting

Beech masting events tend to be highly variable across time, often separated by several years (Cleavitt & Fahey, 2017). However, during the fall of 2013 and 2015, Bartlett Experimental Forest experienced two masting events that were interceded, in the fall of 2014, by an extremely low beechnut crop. Masting is driven by climatic variables and is synchronized across regions, even at locations separated by up to 1,000 km (Koenig & Knops, 1998; Piovesan & Adams, 2001). Data from nearby Hubbard Brook Experimental Forest (about 40 km away and at similar elevations to our sites) confirmed our observations at Bartlett Experimental Forest and indicated that beechnut availability was ~12 times higher during 2013 and 2015 (39.1 and 33.1 seeds/m^2^, respectively) than during 2014 (2.4 seeds/m^2^; Cleavitt & Fahey, 2017). During masting years, nuts are cached by *Peromyscus* species (Figure 1c; Wolff, 1996b) and consumed through the summer of the following year. As such, our high mast seasons were summer 2014 and fall 2015, whereas our low mast seasons were fall 2014 and summer 2015. Although beech trees were not distributed evenly among hardwood, mixed, and softwood forest types (average basal area [m^2^/ha] of 13.4, 2.9, and 1.9, respectively), all grids contained trees capable of producing mast (Leak & Graber, 1993), with at least 12 beech trees/ha that were ≥10 cm in diameter and at least two trees/ha that were ≥30 cm. *Peromyscus* also will travel over 120 m to collect food items and can store over 8 liters of husked beechnuts (Hamilton, 1941), further suggesting that beechnuts were available to individuals on all grids.

Other common mast‐producing trees in our study area included eastern hemlock, red spruce, and red maple. At Bartlett Experimental Forest, eastern hemlock and red spruce masted in the fall of 2013 and 2015 and red maple masted in the spring of 2014 and 2015. Based on data from Hubbard Brook, red maple seeds were over five times more abundant during the summer of 2015 compared to the summer of 2014 (Nick Rodenhouse; personal communication). Despite masting of these tree species, relative to beechnuts, their seeds are likely not a preferred food source. Rodents select large seeds that are energy‐rich, nitrogen‐rich, and easy to collect (Jensen, 1985). Relative to seeds from other tree genera, beechnuts have more calories per gram (Grodziński & Sawicka‐kapusta, 1970; Jensen, 1985) and are easy to collect as they are concentrated near the tree trunk from barochory dispersal (gravity), rather than scattered across the forest floor by the wind (Hughes, Fahey, Hughes, & Torrey, 1988; Wagner et al., 2010). Additionally, excluding the inedible seed coats, beechnuts on our grids (mean ± *SD* of 175.3 ± 37.2 mg, *n* = 36) were over 20 times larger than red maple seeds (8.5 ± 1.9 mg, *n* = 75) and nearly 80 times larger than red spruce seeds (2.3 ± 0.5 mg, *n* = 25) or eastern hemlock seeds (2.2 ± 0.5 mg; *n* = 25).

### 2.3 Stable isotope measurement

Hair samples were soaked in 2:1 chloroform:methanol for 24 hr to remove surface oils, after which they were rinsed, air dried, and cut into small pieces. Food items were rinsed with 2:1 chloroform:methanol and ground to a fine powder. Hair samples (1 mg) and food items (1–5 mg) were weighed into tin capsules and analyzed for stable carbon (δ^13^C) and nitrogen (δ^15^N) isotopes and elemental composition (%C, %N) at the University of New Hampshire Stable Isotope Laboratory using an Elementar Americas Pyrocube elemental analyzer coupled to a GeoVision isotope ratio mass spectrometer. Raw δ^13^C and δ^15^N values were adjusted based on a 3‐point normalization using in‐house standards of sorghum flour, Atlantic cod, and black spruce needles. Isotopes are expressed in delta (*δ*) notation as parts per thousand (‰) deviation from the standard using the formula:

$$\delta =\left[\left(R_{\text{sample}}/R_{\text{standard}}\right)-1\right]\times 1,000.$$

where *R* is the ratio ^13^C/^12^C or ^15^N/^14^N, and standards are Vienna Pee Dee Belemnite (δ^13^C) and atmospheric N_2_ (δ^15^N). Measurement precision based on repeated analyses of in‐house standards was ±0.1‰ for δ^13^C and ±0.2‰ for δ^15^N. To capture an isotopic signal of the general population and avoid an individual grid from biasing our results, we used up to nine hair samples per species from a grid within a season.

### 2.4 Stable isotope integration period and values

Unlike other animal tissues (e.g., muscle or liver) that continuously turn over, hair is metabolically inactive and integrates an isotopic signature of diet at the time of growth (Dalerum & Angerbjörn, 2005). Thus, the isotopic signature of diet assimilated by hair may be offset from the collection time and an understanding of molting ecology is required to determine the temporal window of integration (Fraser, Longstaffe, & Fenton, 2013). In *Peromyscus* spp., individuals go through both ontogenetic and seasonal molts. Young‐of‐the‐year molt from juvenile to subadult pelage and again from subadult to adult pelage. Depending on the time of birth, these ontogenetic molts take place either during the summer or fall and can take as little as 10 days to complete (Gottschang, 1956; Tabacaru, Millar, & Longstaffe, 2011). Adult *Peromyscus* typically have two seasonal molting periods, one in early summer following the breeding season and one in the fall (Brown, 1963; Tabacaru et al., 2011). Because ontogenetic molts are characterized by changes in hair color and seasonal molts by changes in both hair color and hair length (Collins, 1923), we could bin hair samples into distinct summer (11 week period from May 15 to August 7) and fall (11 week period from August 8 and October 31) seasons for both years. Methods used to construct bins are detailed in Appendix S1. To verify that these seasonal bins captured shifts in diet, we compared δ^13^C and δ^15^N values of hair samples collected from individuals (*n* = 47) that were recaptured in multiple seasons. For both *Peromyscus* spp., these individuals showed marked shifts in isotopic values among seasons that closely matched the magnitude and spread of both δ^13^C and δ^15^N values of the general population (see Figure S1). For young of the year, with two hair samples collected during the same seasonal bin, we randomly selected one of the samples to include in analyses. We excluded hair samples from juveniles (≤9 g) because they likely reflect their mother's milk rather than freely consumed food sources (Miller, Millar, & Longstaffe, 2011).

Seasonal bins are appropriate because hair records an isotopic signature of diet during hair growth and molting peaks in the early summer and fall. However, molting can be somewhat individualistic, especially for adults that may not go through a summer molt (Tabacaru et al., 2011; see Figure S1). In some instances, young of the year also may have grown hair spanning both the summer and fall seasons. To ensure that hair samples from adults were not assigned to the wrong season and that subadults incorporating a diet signal across seasons did not influence our analyses, we identified and removed outliers within a season that indicated a mismatch in the diet signal compared to the rest of the population. We checked for multivariate outliers of δ^15^N and δ^13^C using the adjusted quantile method of “aq.plot()” in the R package “mvoutlier” (Filzmoser & Gschwandtner, 2017; R Development Core Team, 2016). This resulted in the removal of five *P. leucopus* (2.9%) and six *P. maniculatus* (3.2%), which did not alter the overall patterns in niche breadth and overlap we observed between the two species (Figure S2).

Prior to our niche analyses, we identified factors influencing δ^15^N and δ^13^C values (and their spread) using linear mixed effects models in “lme” from the R package “nlme” (Pinheiro, Bates, DebRoy, & Sarkar, 2012; Zuur, Ieno, Walker, Saveliev, & Smith, 2009). This allowed us to determine if there were shifts in δ^15^N or δ^13^C values associated with masting phase and whether forest type should be considered in further analyses. Fixed effects included species (*P. leucopus* and *P. maniculatus*), season (summer and fall), masting phase (masting and nonmasting), and forest type (hardwood, mixed, and softwood). For random effects, we considered both a random intercept of year (to account for between‐year variation) and grid within year (to account for between‐grid variation within a year). For spread, we considered a multiple variance structure that allowed residual error to vary by season, masting phase, or forest type. The random intercept (year or grid nested within year) and the multiple variance components were sequentially compared with the final random effects structure selected using Akaike's information criterion (AIC) and a likelihood ratio test. Model fit was assessed by plotting residuals versus fitted values and by evidence of homogeneity of variances and normality of both the residuals and random effects (Zuur et al., 2009).

### 2.5 Dietary composition and niche analyses

Our mixed effects models indicated that forest type had no significant effect on δ^15^N or δ^13^C values of hair (see results), and preliminary analyses confirmed similar patterns among forest types. Therefore, we combined forest types for all analyses, giving a sample size of ≥24 for all groups, which is recommended for robust isotopic niche estimates and reduced uncertainty surrounding them (Syväranta, Lensu, Marjomäki, Oksanen, & Jones, 2013).

We assessed the diets of *P. leucopus* and *P. maniculatus* using Bayesian stable isotope mixing models in the R package “MixSIAR” (Stock & Semmens, 2013). *MixSIAR* uses δ^15^N and δ^13^C values from both consumer tissues (i.e., hair) and each food source along with discrimination factors, elemental concentrations, and the uncertainties surrounding those values to calculate the relative proportion of food sources consumed. We used separate models for species in each season and year; running each model with three chains for 200,000 iterations, removing the first 50,000 and thinning by a factor of 50, resulting in 9,000 draws of the posterior distribution.

**Figure 3:**
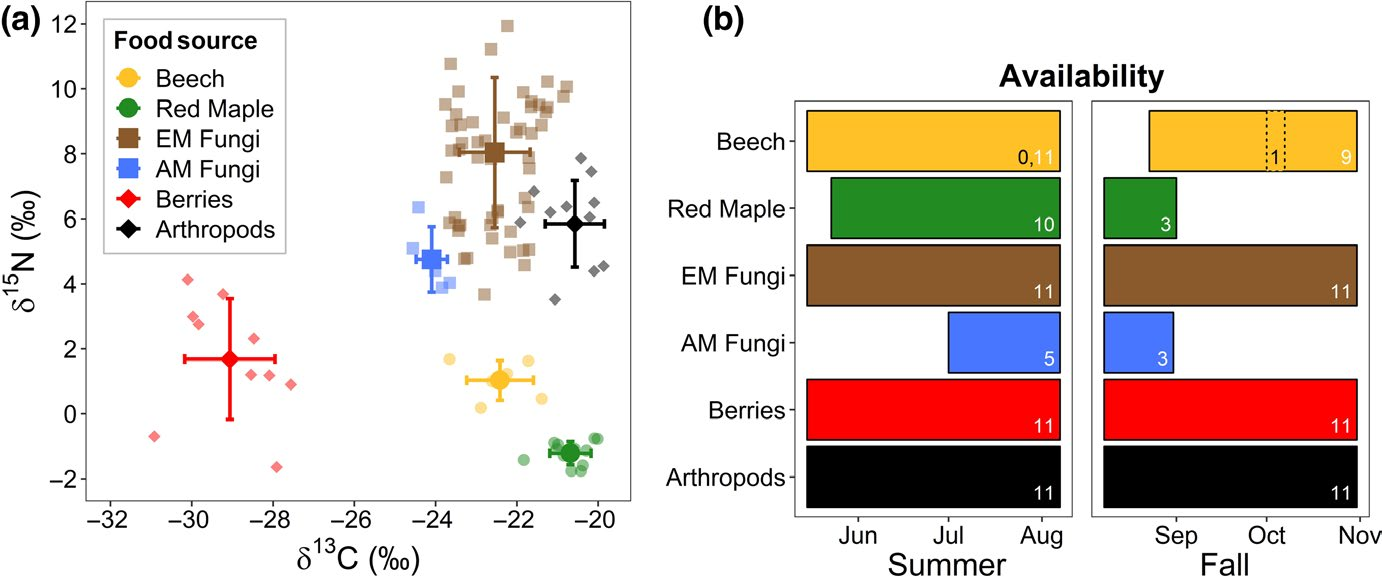
Stable carbon (δ^13^C) and nitrogen (δ^15^N) isotope ratios of major food sources for *Peromyscus leucopus* and *P. maniculatus* (a). Small shapes indicate sample values (analyzed individually for EM and AM fungi and aggregated at the grid level for other food sources) and large shapes with bars indicate means and standard deviation of δ^15^N and δ^13^C of food sources. Values of δ^15^N and δ^13^C have been adjusted by +1.98 and +4.73, respectively, to correct for dietary enrichment; putting food sources into the isotopic space of consumer hair. The availability of each food source (measured in weeks) during the summer and fall hair growing periods (b). For beech, solid boxes and white numbers indicate the number of weeks available during a masting event and dotted boxes and black numbers indicate availability during a nonmasting event

For all mixing models, we used informative priors that improve precision and accuracy (Moore & Semmens, 2008). Informative priors (Dirichlet distribution) were given a total weight equal to the number of food sources (*n* = 6) with the prior for each food source (*α*_k_) scaled by the proportion of weeks they were available relative to the number of weeks the other food sources were available during a given season (Figure 3). Temporal availability was based on phenology recorded in the literature (beechnuts and red maple seeds), food sources observed in the field (berries, arthropods, and EM fungi), and through microscopy of scat (AM fungi). During a masting year, we considered beechnuts to be available from the fall mast into the summer of the following year, whereas during a nonmasting year they were only available for a 1 week period in mid‐October (corresponding to peak nut fall) and were unavailable (*α*_k_ prior set to 0.01) the following summer (Leak & Graber, 1993; Wolff, 1996a). Alternate models where beechnuts were available for 1 week (*α*_k_ = 0.11) in the following summer were qualitatively similar (Figure S3) and thus we report values using *α*_k_ = 0.01. Red maple seeds drop during the last week of May through the end of June (Houle, 1994) and are removed within 1–2 months (Myster & Pickett, 1993). EM sporocarps (Stephens et al., 2017), berries (Gervais & Wheelwright, 2007), and arthropods are available year‐round, whereas AM sporocarps are primarily consumed during summer. For the food source parameters of δ^15^N and δ^13^C, we used means and standard deviations of collected food items and accounted for differences in elemental concentrations. We also ran mixing models with uninformative priors to test the influence of informative priors on our results.

**Figure 4:**
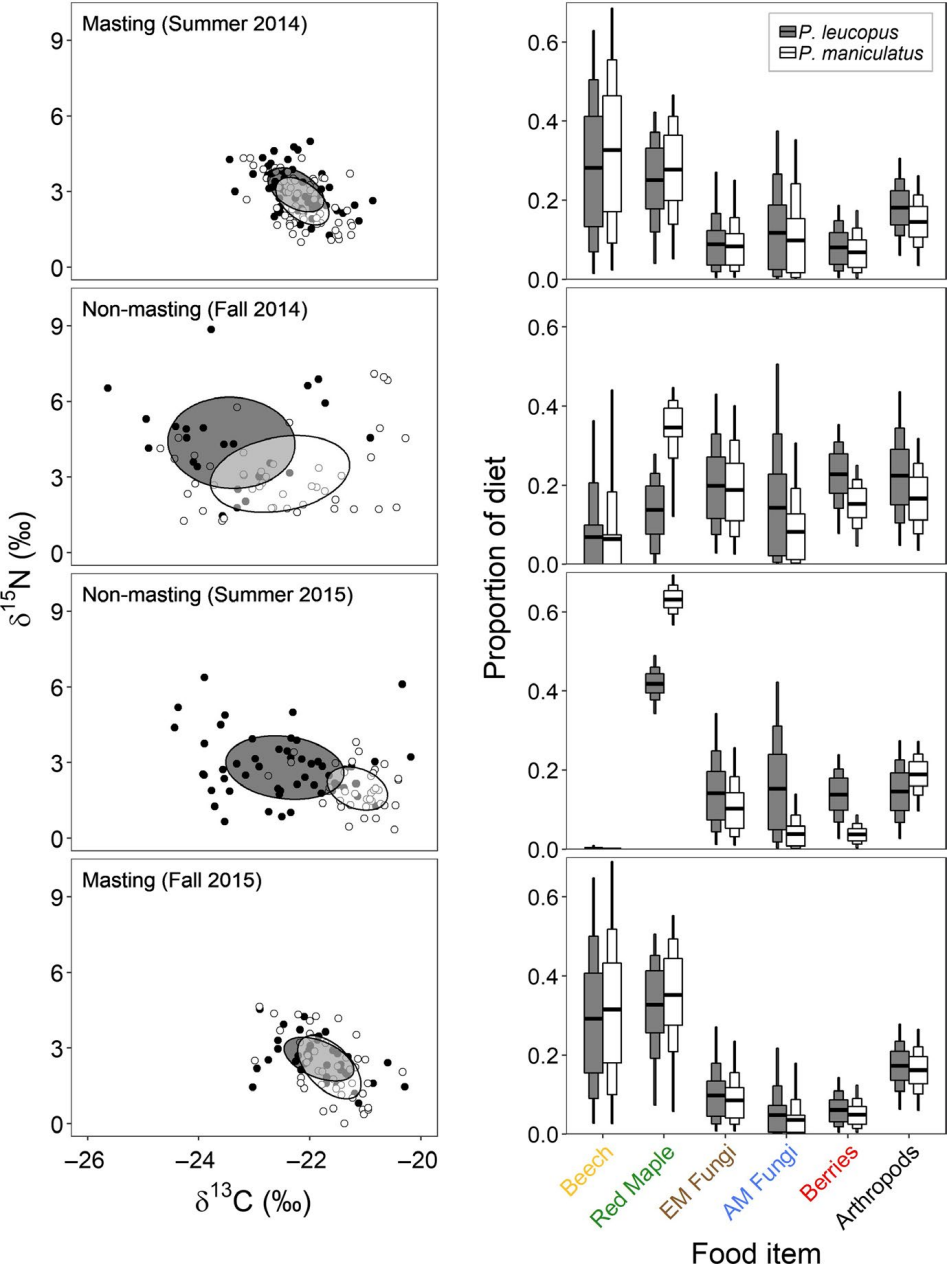
Biplots of stable carbon (δ^13^C) and nitrogen (δ^15^N) isotopes (Left) and results of mixing models showing the proportion of food items contributing to the diets of *Peromyscus leucopus* and *P. maniculatus* (Right) during beech masting and nonmasting phases. Circles represent hair samples of individuals (*P. leucopus* = filled; *P. maniculatus* = open), and ellipses represent the standard ellipse area corrected for small sample size (SEA_c_). Medians of dietary proportions are indicated by a thick horizontal bar, and Bayesian credible intervals are denoted by box width (50% thick box; 75% intermediate box; and 95% thin box). Sample sizes for *Peromyscus leucopus* and *P. maniculatus*, respectively are as follows: summer 2014 (59, 63), fall 2014 (24, 45), summer 2015 (53, 37), and fall 2015 (39, 44)

Consumer tissues are enriched in ^13^C and ^15^N relative to food sources and to put them into consumer isospace they must be adjusted. Although discrimination factors have been derived experimentally for both *P. leucopus* (2.9 for δ^15^N and 1.1‰ for δ^13^C; DeMots et al., 2010), and *P. maniculatus* (3.3 for δ^15^N and 0.3‰ for δ^13^C; Miller, Millar, & Longstaffe, 2008), applying these values to our data caused hair samples to fall well outside the isospace occupied by food sources. Fractionation of both δ^13^C and δ^15^N can vary greatly by diet (Sare, Millar, & Longstaffe, 2005), and it is likely that lab derived discrimination factors from animals fed rodent chow do not reflect those of natural diets. Therefore, we calculated isotopic enrichment factors for natural diets at Bartlett Experimental Forest of 1.98 ± 0.58 for δ^15^N and 4.73 ± 0.37 for δ^13^C using stomach contents (bulk diet) and hair samples from 20 *P. maniculatus* collected by the US Forest Service during the summer of 2015 (for USFS sampling details see Stephens, Burke, Woodman, Poland, & Rowe, 2018). During this time, the diets of *P. maniculatus* were highly constrained (Figure 4), allowing us to minimize effects from intraspecific variation. For bulk diet, stomach contents were adjusted to account for 1‰ enrichment in δ^15^N (negligible for δ^13^C) of stomach contents relative to diet (Hwang, Millar, & Longstaffe, 2007; Sare et al., 2005). Discrimination factors were calculated by subtracting average isotopic values of bulk diet from average isotopic values of hair. Standard error (*SE*) was calculated as ${\left(SE_{\text{stomach}\;\text{contents}}^{2}+SE_{\text{hair}}^{2}\right)}^{1/2}$.

We compared the isotopic niche breadth and overlap of *P. leucopus* and *P. maniculatus* among seasons and years using the R package “SIBER” (Stable Isotope Bayesian Ellipses in R; Jackson, Inger, Parnell, & Bearhop, 2011). Using a Bayesian MCMC algorithm, *SIBER* combines the prior probability with the likelihood of the data to generate a distribution of the covariance matrix to calculate standard ellipse area (SEA; expressed as ‰^2^). The SEA represents the core isotopic niche space occupied by a species and is robust to differences in sample size. We assessed shifts in the shape and location of the isotopic niche breadth and overlap using SEA_c_ (estimated from maximum likelihood and corrected for small sample size). Additionally, we quantitatively compared niche breadth and overlap in each season and year using Bayesian standard ellipse area (SEA_b_) and calculated the probability that the posterior distributions of one group were different from another group. We considered a probability >0.90 to reflect noteworthy differences in the size of the niche breadth or the amount of niche overlap. For both niche breadth and overlap models, we used the same iterative procedures as in the mixing model analysis.

### 2.6 Intraspecific abundance and niche breadth

Our analyses consider the impact of interspecific competition on niche breadth. Intraspecific competition also can broaden population niche breadth when increased abundance increases specialization of individuals within the population (Svanbäck & Bolnick, 2007; Tinker et al., 2012). Populations of *Peromyscus* increase the summer following a fall beech masting event (Conrod & Reitsma, 2015), giving rise to marked year to year variation in abundance. To test for intraspecific effects caused by changes in populations, we assessed the relationship between abundance and niche breadth for each *Peromyscus* species. For abundance, we used the number of unique individuals captured on a grid during the time of molting (i.e., summer or fall). Summer season abundance included individuals captured in June and July. Although we did not sufficiently trap during the fall to assess populations, most reproduction occurs during June and July and it is likely that populations in late summer are similar to those in the fall. Accordingly, fall abundances included captures from July and August. We used *SIBER* to calculate the isotopic niche breadth (SEA_c_) for each grid within a season with at least three hair samples. For each species, we used linear regression to assess the relationship between grid‐level abundance and niche breadth in each season and year.

## 3 RESULTS

More *P. leucopus* and *P. maniculatus* were captured in the masting summer (2014) than in the following nonmasting summer (2015), although the decline was much less dramatic for *P. leucopus* (9.2 ± 5.2–6.8 ± 3.5) than for *P. maniculatus* (30.2 ± 18.6–10.2 ± 6.7). In total, we collected 263 *P. leucopus* hair samples (126 in 2014 and 137 in 2015) and 650 *P. maniculatus* hair samples (455 in 2014 and 195 in 2015). We used up to nine hair samples per species from a grid and season, resulting in 180 *P. leucopus* and 195 *P. maniculatus* hair samples with stable isotope values. After removing multivariate isotopic outliers, sample size included 175 *P. leucopus* (within each season and year: average 43.8; range 24–59) and 189 *P. maniculatus* (average 47.3; range 37–63) hair samples (Figure 4). *Peromyscus* species, season, and masting phase all significantly influenced δ^15^N and δ^13^C values, whereas forest type did not and was dropped from further analyses (Table 1). Masting phase had the largest influence on residual spread of δ^15^N and δ^13^C values, with 3.2 and 4.2 times more variability, respectively, during the nonmasting phase than the masting phase.

**Table 1:** Mixed effects models predicting δ^15^N and δ^13^C values from *P. leucopus* and *P. maniculatus* hair samples

Model components	δ^15^N			δ^13^C		
	*β*	*SE*	*p*‐value	*β*	*SE*	*p*‐value
Fixed effects						
Intercept	3.192	0.573	**<.0001**	−22.449	0.558	**<.0001**
Species (*P. maniculatus*)	−0.510	0.104	**<.0001**	0.306	0.069	**<.0001**
Season (Summer)	−0.702	0.165	**<.0001**	0.646	0.127	**<.0001**
Masting phase (Nonmasting)	0.770	0.149	**<.0001**	−0.806	0.111	**<.0001**
Forest type (Mixed)	−0.285	0.280	.3209	0.100	0.084	.2329
Forest type (Hardwood)	0.325	0.276	.2537	−0.087	0.082	.2911
Random effects						
Year		0.556			0.604	
Grid		0.244			—	
Residual—Masting phase (Masting)		0.654			0.283	
Residual—Masting phase (Nonmasting)		1.905			1.173	

Note

Fixed effects variables included species (*P. leucopus* and *P. maniculatus*), season (summer and fall), masting phase (masting and nonmasting), and forest type (hardwood, mixed, and softwood). For each variable, the effect is relative to the one not listed (e.g., effect of *P. maniculatus* is relative to *P. leucopus*). Random intercept includes year and grid for δ^15^N and year for δ^13^C. Both δ^15^N and δ^13^C models have a residual standard error structure that varies by masting phase. Bolded *p* values denote statistically significant variables at *α* < 0.10.

All potential food sources had distinct δ^15^N and δ^13^C values (Figure 3a). Differences in δ^15^N and δ^13^C between the *Peromyscus* species, season, and masting phase were reflected in the food items consumed (Figure 4). Qualitatively, results of mixing models with uninformative priors were similar to those with informed priors, indicating that prior choice did not drive results of mixing models (Figure S4). During beech masting, the diets of *P. leucopus* and *P. maniculatus* were nearly identical and composed primarily of seeds or nuts with approximately a third coming from beechnuts alone (Figure 4). During nonmasting phases, diets varied widely and beechnuts contributed very little. In the first nonbeech masting season (Fall 2014), *P. leucopus* consumed more berries and arthropods and less red maple than *P. maniculatus*. Overall consumption of both fungi and berries was approximately two times higher during the nonmasting fall of 2014 compared to the masting fall of 2015 for both *P. leucopus* and *P. maniculatus*. During the second nonbeech masting season (summer of 2015), when red maple masted, both species consumed red maple seed, but in different relative proportions; red maple only comprised a third of the diet for *P. leucopus* while it was more than half of the diet for *P. maniculatus*. Additionally, during this period, berries and AM fungi contributed about five times more to the diet of *P. leucopus* than to *P. maniculatus*. Consumption of arthropods was relatively consistent across seasons and years for both *P. leucopus* and *P. maniculatus*.

**Figure 5:**
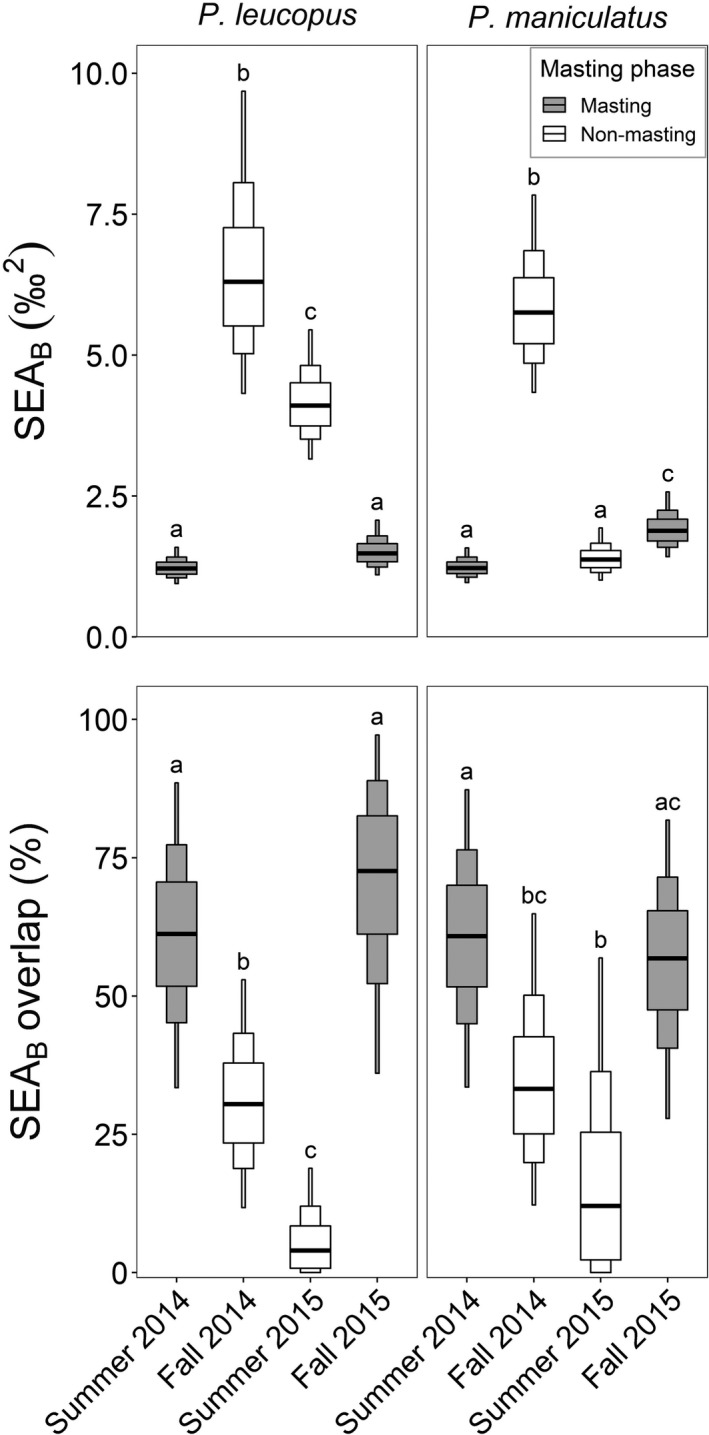
Bayesian standard ellipse area (SEA_B_) (Top) and overlap (Bottom) for *Peromyscus leucopus* and *P. maniculatus* during the masting and nonmasting periods. Medians are indicated by the thick horizontal bar, and Bayesian credible intervals are denoted by box width (50% thick box; 75% intermediate box; and 95% thin box). Different letters between seasons indicate that the probability is >0.90 that the larger group is greater than the smaller group

Changes in the size and location of the isotopic niches of *P. leucopus* and *P. maniculatus* were associated with masting phase and influenced by both δ^15^N and δ^13^C values (Table 1, Figures 4 and 5). For both species, niche breadth (SEA_b_) was generally 3–5 times larger during nonbeech masting seasons (4.1–6.3‰^2^) compared to beech masting seasons (1.2–1.9‰^2^). The exception to this was *P. maniculatus* during the low beech mast summer of 2015 (while red maple seed availability was high) when its SEA_b_ (1.4‰^2^) resembled that of the beech masting summer of 2014 (1.2‰^2^). Despite larger niche breadths during nonmasting seasons, niche overlap (SEA_b_ overlap; 4%–33%) was generally less than half of that observed during beech masting seasons (57%–73%).

Despite variation in intraspecific abundance among trapping grids within seasons, we detected little evidence that increasing population size increased population niche breadth (Figure S5). In fact, for both species, most seasons trended toward the opposite direction with decreasing niche breadth as abundance increased, although this was only significant for the masting summer of 2014 for *P. maniculatus*; Figure S5).

## 4 DISCUSSION

In the absence of manipulative experiments, it can be challenging to assess the roles of foraging ecology and interspecific competition in structuring diet due to the difficulty in quantifying the use of limited resources (Perry & Pianka, 1997; Schoener, 1974). We used natural pulses of beech mast to test the predictions of optimal foraging theory and competition theory for dietary niche breadth and overlap of two closely related rodents that are generalist consumers (*P. leucopus* and *P. maniculatus*). Based on our predictions outlined in the introduction (Figure 2), patterns in niche breadth were generally consistent with optimal foraging theory and patterns in niche overlap consistent with competition theory.

Foraging theory posits that species will select foods that maximize their rate of energy intake. Accordingly, species will have specialized diets when preferred resources are abundant and will broaden diets to include less profitable food items to meet dietary requirements during times of low resource availability (MacArthur & Pianka, 1966; Perry & Pianka, 1997). Our results support optimal foraging theory with a narrowing of the dietary niche breadth and increased consumption of beechnuts for both species during seasons with high beech mast availability. During low beech mast availability, niche breadths generally expanded and consumption of fungi and berries nearly doubled. Although fungi have relatively low nutritional value compared to seeds and require more foraging effort because they need to be extracted from the soil (Cork & Kenagy, 1989), biomass of underground fungal fruiting bodies (truffles) can be extremely high (Stephens et al., 2017). This high abundance likely makes fungi an important component to the diets of *Peromyscus* spp. during times of low seed availability. Berries can also be abundant in the fall and may provide an important source of carbohydrates when seeds are not available. Although we used the most abundant berry‐producing plants on our sampling grids (hobblebush and partridge berry) as an isotopic signature for berries, other understory berry‐producing species, such as Canada mayflower (*Maianthemum canadense*), bunchberry (*Cornus canadensis*), false Solomon's seal (*M. racemosum*), and serviceberry (*Amelanchier* spp.), along with early successional taxa such as pin cherry (*Prunus pensylvanica*) and *Rubus* spp., may also contribute to the diets of the *Peromyscus* spp. We found that arthropods were an important and consistent component to the diets of both *Peromyscus* species. This consumption likely reflected the high protein content of arthropods relative to seeds, fungi, or berries. Thus, arthropods were a complementary resource, supplying protein requirements whereas seeds and berries provided energy (Shaner et al., 2007).

Competition theory predicts that niche overlap between ecologically similar species should be highest when resources are abundant and lowest when resources are limiting, allowing species to coexist during times of low resource availability (Chesson, 2000; Schoener, 1982). Our results support this prediction with high dietary niche overlap during times of beech masting and low niche overlap when beech mast was not available. This dietary plasticity and niche partitioning during times of low food availability may explain why these species often co‐occur with little spatial or habitat segregation (e.g., Wolff, 1985). Similar niche overlap dynamics in response to masting events have been observed in other rodents in Poland and the western United States (e.g., Reid, Greenwald, Wang, & Wilmers, 2013; Selva et al., 2012). Despite considerable investigation into the mechanisms that promote coexistence between *P. leucopus* and *P. maniculatus*, only one prior study has investigated diets of these species in syntopy. Using analysis of stomach contents collected during summer through winter, Wolff et al. (1985) concluded that these species had similar diet habits and likely did not compete for food. Our use of stable isotopes, that integrate diet over an entire season, coupled with comparative data during masting and nonmasting, allowed us to capture niche partitioning that would otherwise be difficult to observe in a field study.

In contrast to the large niche breadths of *P. leucopus* during both seasons of low mast availability, *P. maniculatus* had variable niche patterns during this time. While its niche expanded during the first nonmasting mast period (fall 2014), a response consistent with optimal foraging theory, its niche contracted during the second low mast period (summer 2015), consistent with competition theory. This later niche contraction was during high red maple seed availability and may reflect differences in foraging strategies between the two *Peromyscus* species. Our predictions of niche dynamics assumed that both *Peromyscus* species were dietary generalists and had similar abilities to collect food resources. Experimental feeding trials by Cramer (2014) suggest that *P. maniculatus* may be a seed specialist whereas *P. leucopus* is a generalist, having little preference for seed type. Our findings support this experimental work and suggest that, compared to *P. leucopus*, *P. maniculatus* may have a lower giving‐up density (food density at which an individual stops foraging in a patch) for red maple seeds and can capitalize on this ostensibly lower quality food source when it is highly available. This may be especially true during low population densities when *P. maniculatus* increases foraging time (Davidson & Morris, 2001). Variability in niche response to low food availability has also been documented in other systems. For example, Correa and Winemiller (2014) observed both niche expansion and niche stasis among Amazonian fish in response to reduced terrestrial subsidies. It is likely the complicated interplay between species‐specific foraging behaviors and differences in availability of alternative food sources in natural communities that generates heterogeneity in patterns of niche breadth. During times of low resource availability, species may either expand their dietary niche or specialize on a single food item, depending on how much of a generalist or specialist the species is and the extent to which alternative food sources are available.

Population density can also play a role in niche dynamics, particularly by influencing intraspecific competition. For example, higher densities can lead to decreased food availability, causing phenotypically different individuals to choose different alternative prey that in turn promotes specialization (Svanbäck & Bolnick, 2007). This increased specialization, at the individual level, can lead to an overall larger niche breadth of a population (Svanbäck & Bolnick, 2007; Tinker et al., 2012). Although we cannot directly test the impact of intraspecific competition on niche breadth, abundance and niche breadth were not significantly correlated for either species. In fact, we observed a general pattern of decreased niche breadth with increased abundance, even for *P. maniculatus* that reached very high abundances on some grids during 2014 (Figure S5). In addition to increasing competition, higher densities can decrease home range size (Bogdziewicz, Zwolak, Redosh, Rychlik, & Crone, 2016). Marked differences in abundances among years (higher in masting summer of 2014 compared to nonmasting summer 2015) may have influenced the area over which individuals could forage. These intraspecific impacts may have modulated niche breadth in ways consistent with optimal foraging theory and could have contributed to the magnitude of the response we observed (Schoener, 1971; Stephens & Krebs, 1986).

Spatial resource partitioning between the species may have also influenced dietary niche dynamics. Although *P. leucopus* and *P. maniculatus* do not differ in territoriality or aggression when defending space (Klein, 1960; Wolff, 1985), in some forested systems, they differ in microhabitat or vertical space use (e.g., Barry, Botje, & Grantham, 1984; Parren & Capen, 1985; but see Graves, Maldonado, & Wolff, 1988). Although we did not explicitly measure microhabitat selection in this study, we did capture both species on sampling grids, often at the same station. However, slight differences in microhabitat partitioning could have altered the amount or type of food resources available to individuals, subsequently contributing to differences in diet. This would likely be most apparent during nonmasting times when seed availability is low and other resources (e.g., truffles or berries which are often patchily distributed) are consumed, leading to increased specialization. Thus, similar to intraspecific competition, interspecific microhabitat partitioning may have contributed to the magnitude of the seasonal variation we observed.

Our findings highlight that broad similarity among congeners may mask important differences and caution against assumptions of equivalency in ecological studies (e.g., Conrod & Reitsma, 2015; Schnurr, Ostfeld, & Canham, 2002). Although the diets of both *Peromyscus* converged during times of beech masting, they diverged during seasons with low mast availability. This suggests that competition between these species may alter selection for food resources as a result of underlying differences in foraging efficiencies (e.g., giving‐up‐densities for food items). This diet plasticity may also have important implications for changes in ecosystem function. For example, during nonmasting seasons, consumption of fungal sporocarps nearly doubled. A rise in fungal consumption likely increases spore dispersal of the mycorrhizal fungi that are required for tree growth and seedling establishment (Maser, Trappe, & Nussbaum, 1978). Dietary switching of these generalist consumers could also influence interspecific interactions with other rodents, particularly those that feed on seeds or fungi. For example, woodland jumping mice (*Napaeozapus insignis*), southern red‐backed voles (*Myodes gapperi*), and eastern chipmunks (*Tamias striatus*) are common rodent consumers in northeastern North America that likely compete for food resources with the *Peromyscus* species (Conrod & Reitsma, 2015; Schnurr et al., 2002). The influence of niche partitioning and species interactions on food selection and its cascading effects on niche dynamics and ecosystem function warrant further study.

## CONFLICT OF INTEREST

The authors declare no competing interests.

## AUTHOR CONTRIBUTIONS

RBS and RJR conceived the ideas and designed methodology. RBS collected and analyzed the data and EAH aided with interpretation of the isotopic data. RBS led the writing of the manuscript and all authors contributed critically to the drafts.

## Data Availability

Data supporting this study are available from the Dryad Digital Repository (https://doi.org/10.5061/dryad.77vr729).
