# Supplementary material for: Pulsed resource availability changes dietary niche breadth and partitioning between generalist rodent consumers
Source: Ecol Evol. 2019 Aug 20;9(18):10681–93. doi: 10.1002/ece3.5587 (PMC6787868; doi:10.1002/ece3.5587)
Supplement: Supplementary file 1 [file ECE3-9-10681-s001.docx]

**Electronic Supporting Materials to: Pulsed resource availability changes dietary niche breadth and partitioning between generalist rodent consumers**

Appendix S1

Hair is metabolically inert and integrates an isotopic signature of diet at the time of growth. Because *Peromyscus* go through ontogenetic and seasonal molts at certain times of the year, the window of dietary assimilation by hair may be offset from the time of collection. Both ontogenetic molts and seasonal molts are characterized by distinct changes in hair color and length (Collins, 1923), making it possible to determine the stage of molt an individual was in. In our system, we observed most young-of-the-year going through ontogenetic molt (juvenile to sub-adult pelage and sub-adult to adult pelage) between June and early August. Additionally, most adults had a summer molt in July, after the breading season, and a winter molt from mid-August through the end of October. Below, in Figure S1, we demonstrate the change in carbon (δ^13^C) and nitrogen (δ^15^N) recorded in hair samples from the same individual sampled under different molt phases.

To correctly match hair samples collected during trapping to their time of growth, we constructed two seasonal bins corresponding with the summer season (11 week period from May 15 to August 7) and the fall season (11 week period from August 8 to October 31). For the summer season, the isotopic integration bin starts approximately two weeks prior to when trapping began (June 1) to account for hair from sub-adult individuals that had gone through molt at the start of sampling (Gottschang, 1956). The summer season included hair samples collected from young-of-the-year between June 1 and August 19 (last day of summer sampling) and hair samples collected from adults between July 2 and August 19. The fall season included hair samples from all individuals collected during fall sampling (September 12 through October 3). Additionally, hair samples from adults captured between June 1 and July 1 (prior to summer molt) were assigned to the fall season of the prior year. A small number of adults (*n* =10) captured between July 2 and July 9 had not gone through a summer molt, as indicated by their winter pelage, and were included with the fall season of the prior year. Additionally, several individuals (*n* = 8) were in the process of entering their winter pelage at the end of the summer sampling period and were included in the fall season of that year as the hair was likely grown after August 8.

**References**

Collins, H. H. (1923). Studies of the pelage phases and of the nature of color variations in mice of the genus *Peromyscus*. *Journal of Experimental Zoology*, *38*(1), 45–107. doi.org/10.1002/jez.1400380103

Gottschang, J. L. (1956). Juvenile molt in *Peromyscus leucopus noveboracensis*. *Journal of Mammalogy*, *37*(4), 516–520. doi.org/10.2307/1376646


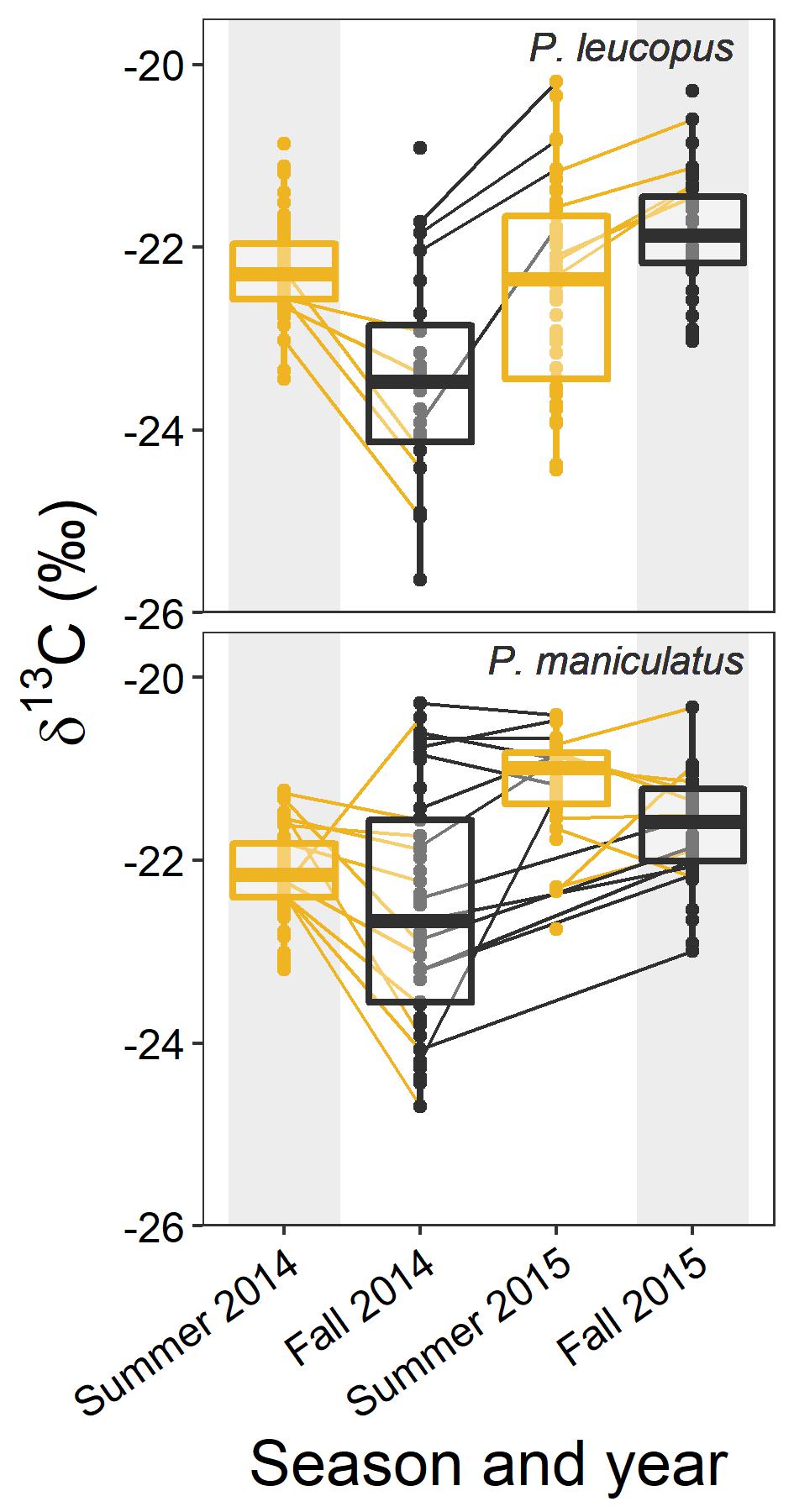

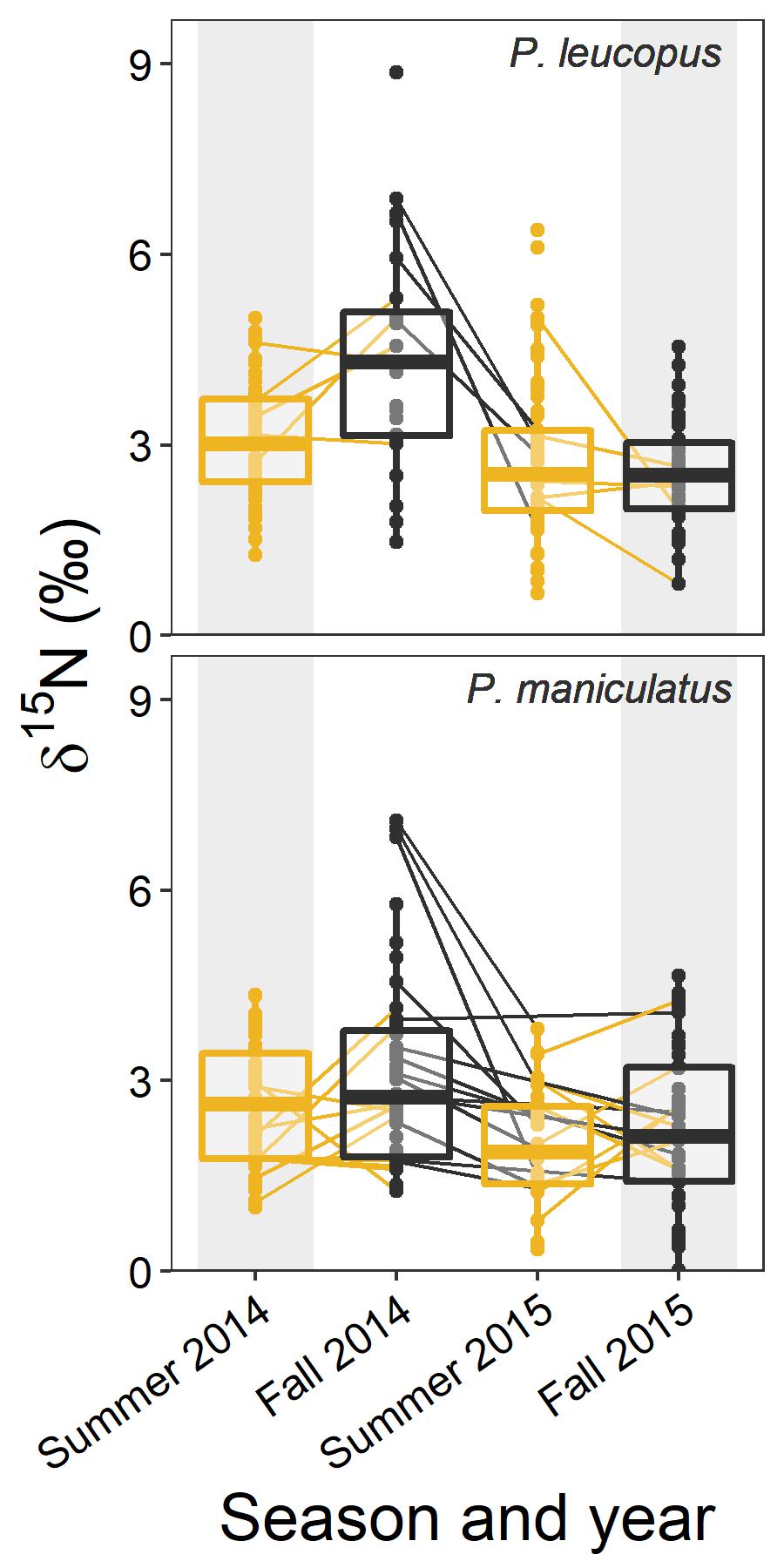


Figure S1. Boxplots of stable carbon (δ^13^C, left) and nitrogen (δ^15^N, right) isotopes from hair samples of individual *P. leucopus* and *P. maniculatus* during seasons with beech mast available (gray vertical bars) and seasons with no beech mast available. Lines connect hair samples from the same individual (i.e., molting occurred with hair grown in different seasons). Note that in some cases *P. maniculatus* (*n* = 6) did not go through a summer molt as indicated by a line directly from Fall 2014 to Fall 2015.


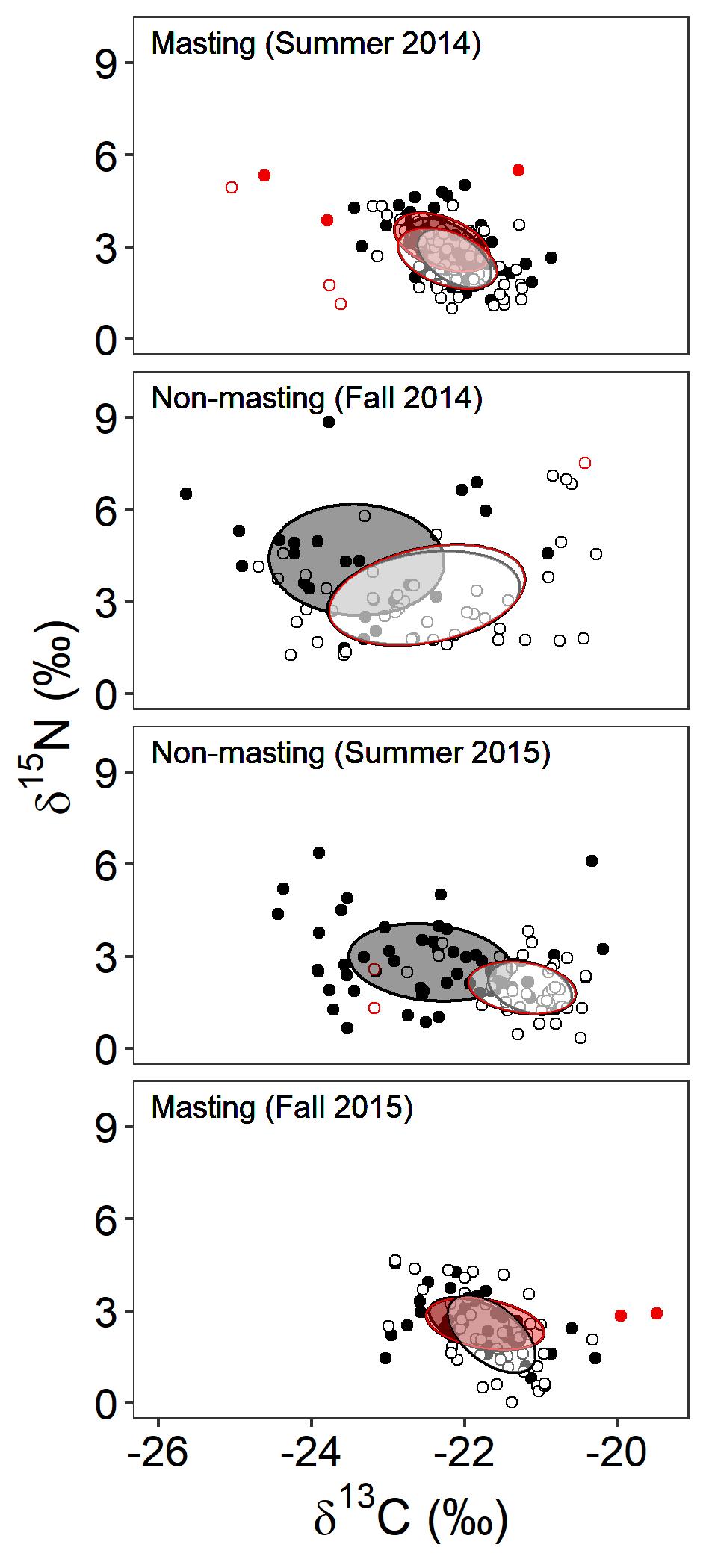


Figure S2. Biplots of stable carbon (δ^13^C) and nitrogen (δ^15^N) isotopes during beech masting and non-masting phases. Circles represent hair samples of individual *P. leucopus* (filled) and *P. maniculatus* (open) with red denoting multivariate outliers. Ellipses represent the standard ellipse area corrected for small sample size (SEA_c_). Gray filled (*P. leucopus*) and white filled (*P. maniculatus*) ellipses were calculated with outliers removed and red filled (*P. leucopus*) and red outlined (*P. maniculatus*) ellipses were calculated using all samples. Only one ellipse is shown for species in seasons with no outliers (*P. leucopus* in the fall of 2014 and summer of 2015 and *P. maniculatus* in the fall of 2015).


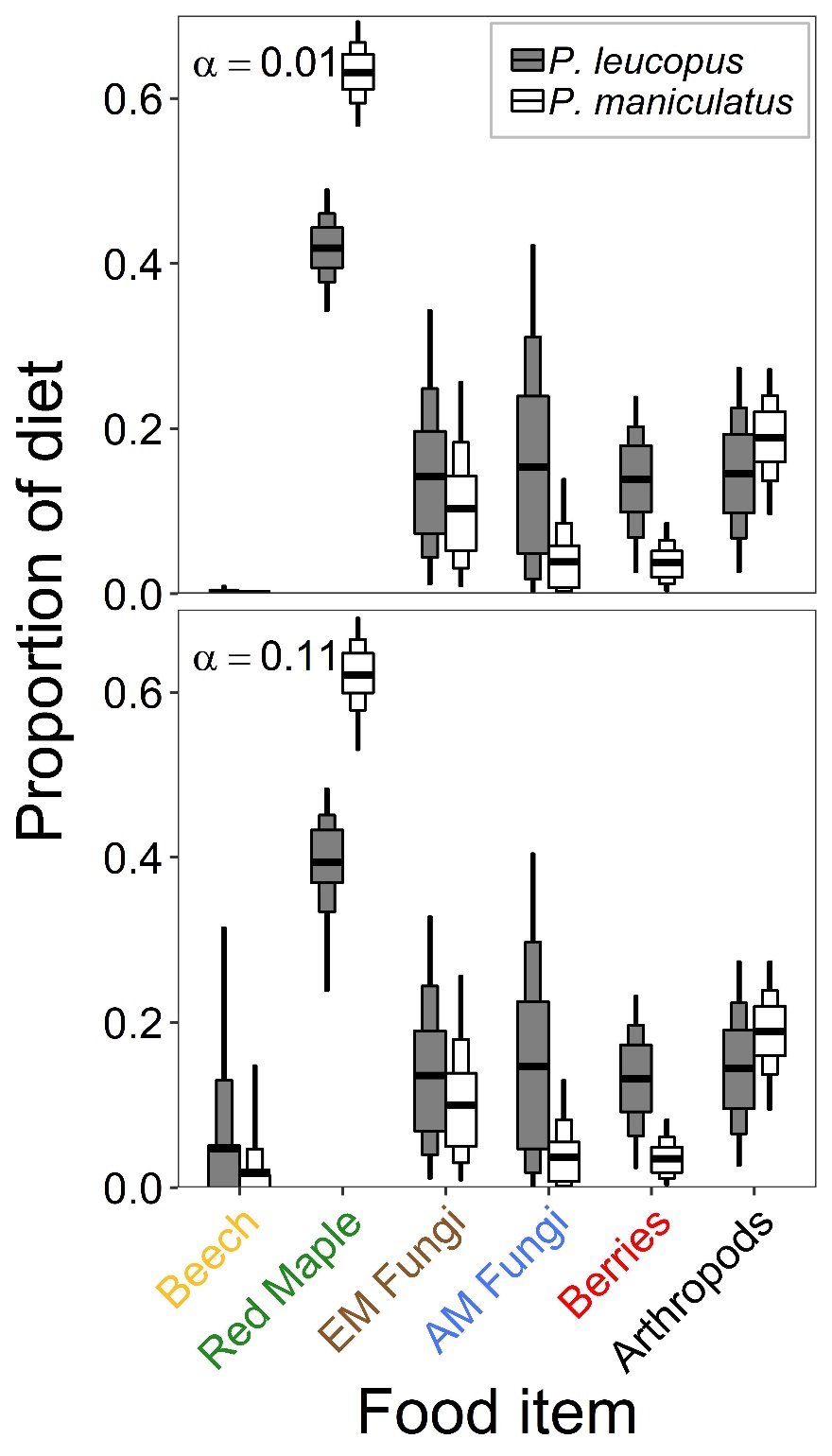


Figure S3. Results of mixing models showing the proportion of food items contributing to the diets of *Peromyscus leucopus* and *P. maniculatus* during the non-masting summer of 2015. Models in the top panel use α = 0.01 to represent beech mast availability and models in the bottom panel use alpha scaled to represent one week of beech mast availability (α = 0.11).


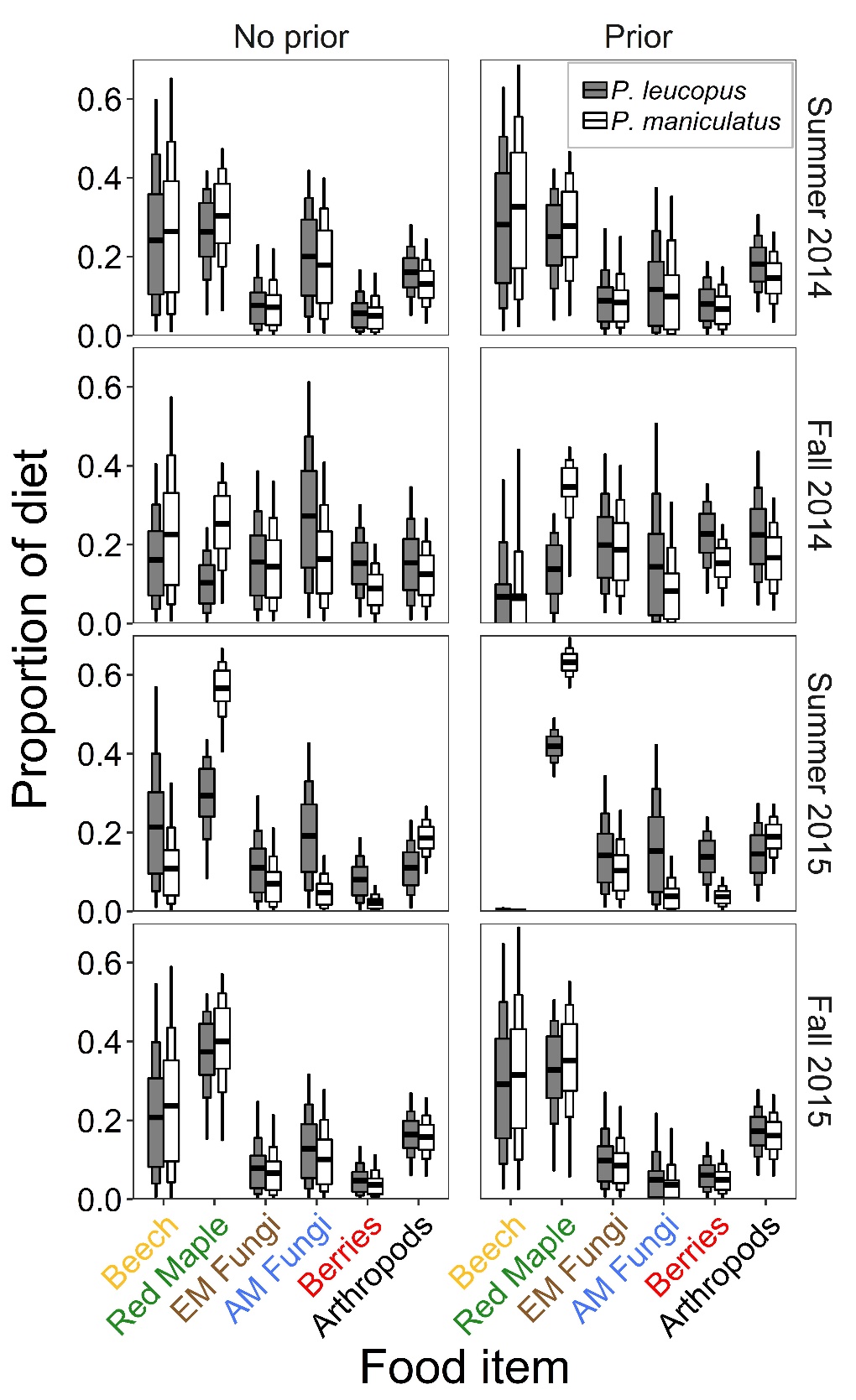


Figure S4. Results of mixing models showing the proportion of food items contributing to the diets of *Peromyscus leucopus* and *P. maniculatus* using uninformative priors (left) and informed priors (right).


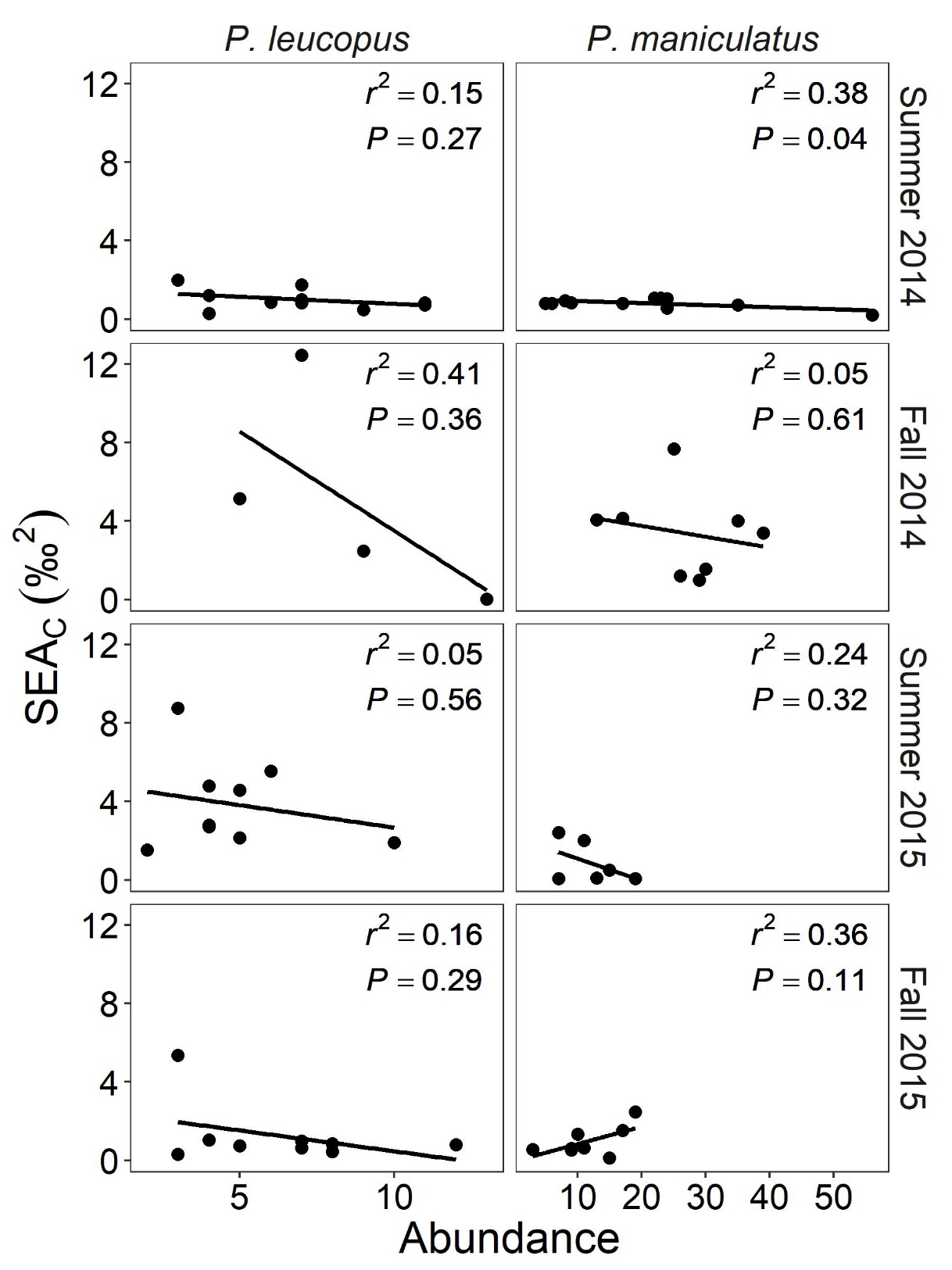


Figure S5. Relationship between intraspecific standard ellipse area corrected for small sample size (SEA_c_; a proxy for niche breadth) and abundance of *P. leucopus* and *P. maniculatus*. Within a season, only grids with at least three hair samples were used to calculate SEAc. Summer 2014 and Fall 2015 were masting seasons and Fall 2014 and Summer 2015 were non-masting seasons for American beech. Regression *r*-squared and *P*-values, for each species within a season and year, are indicated in the upper right corner of each box.
